# Advantages of the Combinatorial Molecular Targeted Therapy of Head and Neck Cancer—A Step before Anakoinosis-Based Personalized Treatment

**DOI:** 10.3390/cancers15174247

**Published:** 2023-08-24

**Authors:** Robert Kleszcz

**Affiliations:** Department of Pharmaceutical Biochemistry, Poznan University of Medical Sciences, 4, Święcickiego Str., 60-781 Poznan, Poland; kleszcz@ump.edu.pl

**Keywords:** head and neck cancer, molecular targets, chemotherapy, combinatorial therapy, HPV, EGFR, PI3K, signaling pathways, cancer stem cells, anakoinosis

## Abstract

**Simple Summary:**

Head and Neck Squamous Cell Carcinoma (HNSCC) is a major threat to public health around the world. Its occurrence is linked to genetic events and environmental factors, including Human Papilloma Virus (HPV) infections. Patients with HPV-positive tumors usually have a better prognosis than those with HPV-negative tumors. According to advances in understanding the molecular basis of HNSCC tumors, targeted therapy is thought to improve treatment outcomes. This article discusses the most important molecular targets for HNSCC and primarily demonstrates various perspectives on combinatorial molecular targeted therapy. Disruption of cancer cell signaling and microenvironmental homeostasis, targeting epigenetic modulators, energy metabolism, or oxidative stress are all common elements in anakoinosis-based therapy, which is a therapy that targets cancer cell intercellular and intracellular communication. Thus, those concepts have been described as potential ways to improve the prognosis of HPV-negative patients.

**Abstract:**

The molecular initiators of Head and Heck Squamous Cell Carcinoma (HNSCC) are complex. Human Papillomavirus (HPV) infection is linked to an increasing number of HNSCC cases, but HPV-positive tumors generally have a good prognosis. External factors that promote the development of HPV-negative HNSCC include tobacco use, excessive alcohol consumption, and proinflammatory poor oral hygiene. On a molecular level, several events, including the well-known overexpression of epidermal growth factor receptors (EGFR) and related downstream signaling pathways, contribute to the development of HNSCC. Conventional chemotherapy is insufficient for many patients. Thus, molecular-based therapy for HNSCC offers patients a better chance at a cure. The first molecular target for therapy of HNSCC was EGFR, inhibited by monoclonal antibody cetuximab, but its use in monotherapy is insufficient and induces resistance. This article describes attempts at combinatorial molecular targeted therapy of HNSCC based on several molecular targets and exemplary drugs/drug candidates. The new concept of anakoinosis-based therapy, which means treatment that targets the intercellular and intracellular communication of cancer cells, is thought to be the way to improve the clinical outcome for HNSCC patients. The identification of a link between molecular targeted therapy and anakoinosis raises the potential for further progress in HPV-negative HNSCC therapy.

## 1. Introduction

Cancer is a complex group of diseases that is an enormous burden on the health of the world’s population. In 2020, there were about 19.3 million new cancer cases and nearly 10 million cancer deaths [1]. Head and Neck Squamous Cell Carcinoma (HNSCC) contributes significantly to these statistics. HNSCC tumors develop in the mucosal linings of various locations in the head and neck, most notably, the oral cavity, lip, nasopharynx, oropharynx, hypopharynx, and larynx [2]. HNSCC is the sixth-most-common cancer globally, with mortality rates as high as 40–50% (over 300,000 annual deaths worldwide), making it a serious oncological problem [1,3,4]. Head and neck cancers are frequently classified based on the presence of the Human Papillomavirus (HPV), as observed, for example, in clinical trials based on HPV status [5].

Tobacco and alcohol use are commonly associated with the development of HPV-negative HNSCC. Tobacco smoke contains approximately 9500 chemicals, including 80 known carcinogens [6]. Furthermore, smokeless tobacco (for chewing as tobacco leaves or as products for oral and nasal use) contains tobacco-specific nitrosamines, N-nitrosamine acids, volatile N-nitrosamines, aldehydes, and other carcinogenic compounds, such as heavy metals [7]. High-proof alcohol consumption is known to synergize with tobacco use to promote carcinogenesis in HPV-negative HNSCC because alcohol may act as a solvent for tobacco-derived carcinogens, increasing epithelial cell exposure to those chemicals [8]. Environmental risk factors for HPV-negative HNSCC development, like insufficient oral cavity hygiene generating inflammation or highly processed food, pose a severe threat [9]. Mutations in the *TP53* gene, which encodes the tumor suppressor protein, p53, are the most common in HPV-negative tumors, followed by the *CDKN2A* gene, which encodes p16, an inhibitor of cyclin-D1-dependent kinase 4/6 (CDK4/6). Lack of p53 and p16 activity disrupts cell cycle control, for example, in the latter case by increased phosphorylation and inactivation of the pRb protein [10].

Head and neck cancers caused by HPV infections primarily affect the oropharynx [11]. HPV-16 is the dominant virus type with a high risk of HNSCC development after infection, which is caused less frequently by HPV-18 and very rarely, by other HPV-types such as HPV-33 and HPV-52 [12]. Unlike in HPV-negative HNSCC, HPV-derived oncoproteins, not mutations, are responsible for p53 and pRb protein inactivation in HPV-positive cases. The E6 oncoprotein binds to both E6-associated protein and p53, promoting ubiquitination and degradation of the latter, whereas the E7 oncoprotein ubiquitinates pRb [13].

HPV-negative and HPV-positive head and neck tumors have distinct characteristics. Somatic mutations in cancer genomes result from various molecular processes, each creating a characteristic mutational signature [14,15]. HPV-negative tumors of the head and neck present mainly smoking-related mutational signatures, represented by SBS4 (single-base substitution 4), with a large proportion of C > A mutations and DBS2 (doublet-base substitutions 2), composed predominantly of CC > AA mutations [16,17]. In turn, HPV-mediated cancers are typically dependent on the apolipoprotein-B mRNA-editing, catalytic-polypeptide-like (APOBEC) family of cytidine deaminases’ mutation signature, including SBS2 and DBS11 in HPV-positive HNSCC [14,18,19,20]. The HPV-derived E6 oncoprotein promotes the expression of APOBEC3B [21].

The mean age at HNSCC onset in HPV-positive patients was previously much lower than in HPV-negative HNSCC patients, but this difference has recently narrowed [22]. Based on 3-year overall survival rates, patients with HPV-dependent tumors have a better prognosis than patients with HPV-negative cancer, with 82.4% vs. 57.1%, respectively [23]. Although HPV-positive incidents are rising, in tandem, their treatment response has also improved, which is not observed for HPV-negative HNSCC [24]. Thus, because of the poor prognosis in HNSCC that develops independently of HPV infections, especially in recurrent and metastatic cases, finding effective treatment options is a current priority.

The standard treatments for HNSCC are surgery and/or radiotherapy. For oral cavity cancers, surgery is commonly used, whereas radiation may be more appropriate for pharyngeal and laryngeal cancers [9]. Chemotherapy-based treatment should be implemented in patients with advanced tumors or difficult localizations. Unspecific chemotherapeutics block the cell cycle and induce mechanisms of cell death. However, the treatment of modern oncological diseases must target other features of tumors in order to result in better outcomes. HPV-negative tumors have a high mutation rate and chromosomal aberrations, with or without copy number alteration profiles [25]. As a result, many molecular targets for cancer cell features were described for head and neck cancers.

This review aims to discuss current perspectives on combinatorial targeted therapy for HNSCC based on the simultaneous inhibition of multiple molecular targets. Such a strategy could be more effective in improving outcomes in patients with HPV-negative head and neck tumors. This therapy concept partly reflects the term, “Anakoinosis,” which will be discussed later. The following section will show an overview of molecular targets, along with examples of drugs/drug candidates that have been tested individually and in combination with standard treatment procedures.

## 2. Molecularly Targeted HNSCC Therapy

### 2.1. Epidermal Growth Factor Receptor (EGFR) Pathway

The most important member of the receptor tyrosine kinases (RTK) family is the epidermal growth factor receptor (EGFR). Cetuximab, a chimeric IgG1 monoclonal antibody against EGFR, was first approved by the U.S. Food and Drug Administration (FDA) in 2004 for patients with irinotecan-resistant colorectal cancer [26], and two years later was authorized for the treatment of locally advanced HNSCC [27]. Even 90% of HNSCC patients have overexpression of the EGFR [28]. Unfortunately, cetuximab has only shown a 20% positive response rate in patients with HPV-negative tumors and only a marginal improvement in combination with radiotherapy and platinum-based chemotherapy [29,30]. Panitumumab is another monoclonal antibody against EGFR [31].

Irreversible EGFR inhibitors act on the intracellular domain, inhibiting the cytoplasmic tyrosine kinase domain. Erlotinib is the most respected representative, and was, for instance, combined with standard docetaxel/cisplatin chemotherapy for recurrent/metastatic (R/M) HNSCC [32]. The combined treatment achieved a response rate of 62% (8% complete response and 54% partial response), which is greater than the previous trial’s response rate of 40% for docetaxel/cisplatin chemotherapy.

Many studies have found that HNSCC and other EGFR-dependent tumors are resistant to EGFR-inhibitory therapy. Yamaoka et al. (2017) summarized four general mechanisms of anti-EGFR antibody and EGFR tyrosine kinase inhibitor resistance in cancer cells: (i) secondary mutations in the *EGFR* gene; (ii) resistance to apoptotic cell death; (iii) phenotypic transformation (e.g., tumor cells activating stem cell-like characteristics); and finally, (iv) activation of alternative signaling pathways [33].

The active state of EGFR triggers a cascade of intracellular responses. As a result, in many cases of molecular abnormalities, even effective EGFR attenuation cannot influence the downstream activation of altered signal transduction elements. Therefore, there is a recurrence of a tumor that is resistant to previously used therapeutic procedures.

### 2.2. Farnesylation of RAS

RAS is a key player in the EGFR signal transduction. The *HRAS* mutations are called “undruggable”, but advances in the high-resolution understanding of RAS isoform structure provide hope for developing personalized therapies for patients with RAS-dependent cancers [34]. Fortunately, post-transcriptional farnesylation is required for RAS protein to be anchored to the inner side of the cell membrane, which is crucial for EGFR signal transduction. A phase II clinical trial of tipifarnib (inhibitor of farnesyltransferase) involving 30 patients with R/M HNSCC revealed positive response in patients with *HRAS* mutations [35].

EGFR-dependent RAS activation stimulates two critical intracellular signaling pathways, RAS/RAF/MAPK and PI3K/Akt/mTOR [36].

### 2.3. RAS/RAF/MAPK Pathway

In brief, this pathway creates kinase cascades and finally activates extracellular signal-regulated kinases (ERK) [37], which translocate from the cytoplasm to the nucleus to induce specific genes expression [38]. In HNSCC, attempts were made to target this kinase cascade by inhibiting the RAF and MEK proteins. For instance, Sorafenib—a RAF kinase, vascular endothelial growth factor receptor (VEGFR) and platelet-derived growth factor receptor (PDGFR) inhibitor [39]—was evaluated in phase II clinical trial of patients with R/M HNSCC and resulted in a partial response or disease stabilization in 40.7–51% of patients [40,41].

### 2.4. PI3K/Akt/mTOR Pathway

Phosphoinositide 3-kinase (PI3K) class IA comprises the p110α/β/δ catalytic subunit and the p85 regulatory subunit. Phosphatidylinositol 3,4,5-trisphosphate, converted from phosphatidylinositol 4,5-bisphosphate, activates downstream signaling factors such as Akt. Another kinase, the mammalian target of rapamycin (mTOR), is the main effector of Akt kinase [42]. Mutations in the PI3K catalytic subunit p110α are the most common genetic abnormality observed in HNSCC. Alpelisib (NVP-BYL719) is the first FDA-approved p110α inhibitor for the treatment of hormone receptor-positive, HER2-negative, PI3K catalytic subunit alpha (*PIK3CA*)-mutated, advanced or metastatic breast cancer, and it may be useful in HNSCC as well [43,44].

Akt phosphorylates a variety of targets, including tuberous sclerosis complex 2 (TCS2), which, along with TCS1, inhibits the activity of the mTOR complex (mTORC) [45]. Based on the U.S. National Library of Medicine online (https://clinicaltrials.gov) database of clinical studies, Akt inhibitors-ipatasertib (GDC-0068) and capivasertib (AZD5363), are tested for R/M HNSCC in mono-treatment (NCT02465060 and NCT02465060, respectively), followed by ipatasertib in combination with cisplatin and radiotherapy (NCT05172245).

Finally, mTOR inhibition may be used to target this pathway, e.g., by everolimus. A meta-analysis of studies involving mTOR inhibition confirms that monotherapy cannot improve the prognosis of HNSCC patients but can accelerate partial tumor response when combined with other anticancer agents [46].

### 2.5. Other Receptor Tyrosine Kinases and Their Downstream Signaling Pathways

Other RTK, in addition to EGFR, may be promising pharmacological targets in HNSCC. In some tumors, the fibroblast growth factor receptor (FGFR) is overexpressed and partially amplified [47] and was linked to poor overall survival and disease-free survival in HPV-negative patients. The small molecule, AZD4547, is a potential FGFR inhibitor, which was found to decrease the growth of HNSCC cells in vitro [48].

The VEGFR signaling orchestrates neovascularization of growing tumors [49]. Because RAS, PI3K, and STAT3 proteins are downstream effectors of VEGFR [50], its simulation promotes many other tumor-promoting features controlled by those pathways. Bevacizumab, a humanized monoclonal antibody against VEGF, is frequently examined in clinical trials; for instance, it was combined with EGF-receptor-targeted therapy based on cetuximab [51] or erlotinib [52], which benefits patients.

The PDGFR signaling, among others, influences Akt-dependent activation of pro-oxidative NF-κB signaling [53]. Overexpression of PDGF and its receptor has been associated with neck lymph node metastasis, advanced TNM stage, and poor survival in HNSCC patients [54]. Multifunctional kinase inhibitors are currently being used to target this receptor along with other RTKs. Imatinib, a PDGF(R) and VEGF(R) inhibitor suppressed their expression synergistically in vitro [55].

In HNSCC, the hepatocyte growth factor/mesenchymal-epithelial-transition factor (HGF/c-MET) pathway promotes PI3K/Akt, RAS/MAPK, STAT3, and Src/NF-κB intracellular signaling, resulting in cancer cell proliferation and apoptosis avoidance, followed by extensive growth and metastasis [56,57]. Wang et al. (2021) used three c-Met inhibitors (crizotinib, tivantinib, and cabozantinib) in combination with the pan-HER inhibitor afatinib. In HNSCC cell lines, xenografts, and patient-derived xenograft animal models, the drugs’ combination exceeds monotherapy regarding anticancer efficacy, confirming the significance of further clinical trials [58].

STAT canonical signaling can be activated by RTK, as described in this section, resulting in neovascularization, increased cell proliferation, survival, and even immune response evasion [59]. The nuclear accumulation of phosphorylated STAT3 has been identified as a prognostic marker in the early premalignant stages of HNSCC [60]. STAT5 inhibitor 573108, in combination with radiotherapy, was found to improve cell survival in a panel of HNSCC cell lines [61].

### 2.6. Cancer Stem Cell-Related Signaling Pathways

Cancer stem cells (CSC) are a subpopulation of cells that express specific extracellular and molecular markers and can self-renew [62,63,64]. After temporary tumor bulk reduction, conventional anticancer therapy that does not affect CSC leads to tumor recurrence with an enriched, therapy-resistant CSC population.

The NOTCH pathway regulates body pattern formation, cell fate, and proliferation during embryogenesis, and stem cell activity in both early and adult organisms [65]. The global mutation rate of *NOTCH1* is approximately 15%, making this gene one of the most frequently mutated in HNSCC [66]. The *NOTCH1* gene was thought to be a tumor suppressor due to the high percentage of mutations in HNSCC [67], but this pathway can be induced in tumors as well [68]. The NOTCH pathway promotes the self-renewal capacity of HNSCC cells, as evidenced by increased expression of Oct4, Sox2, and CD44 stemness markers [69].

The Wnt/β-catenin signaling is essential for cell differentiation and proliferation during embryogenesis and in proliferative tissues in adulthood, including the stem cell subpopulation [70,71]. This signaling is extensively activated in colorectal cancers, but its dysregulation at various levels of signal transduction is also critical for the development of HNSCC [72]. In particular, the porcupine inhibitor (IWP-2) and the inhibitor of the interaction between β-catenin and the CREB binding protein (PRI-724) effectively inhibited HNSCC cell lines [73].

The Hedgehog (Hh) canonical pathway is activated by the Sonic Hedgehog (SHh) ligand and is present in various tissues/organs during development and in the adult organism [74,75]. The significance of the Hh pathway in the development of basal cell carcinoma of the head and neck was practically confirmed by the FDA’s approval of the Hh signaling inhibitor, vismodegib, in 2012 [76]. Several studies [77,78,79,80] have identified active Hh signaling as a negative prognostic marker for HNSCC patients and multi-drug resistance. Furthermore, in HNSCC, Hh signaling is strongly linked to CSC markers [81].

NOTCH signaling activation can upregulate components of the Wnt and Hh pathways, and further crosstalk between those signaling pathways supports the maintenance and development of HNSCC by promoting the activity of CSC [82]. In addition, it is possible that also the Hippo pathway, which is involved in organ development, regeneration, and stemness, could be used as a target for HNSCC combinatorial therapy, while its crosstalk with NOTCH, Wnt, and Hh signaling has been demonstrated [83,84]. Finally, because transforming growth factor-β (TGF-β) is a regulatory cytokine involved in the control of CSC and immune cells [85,86], it is a good target for innovative combinatorial HNSCC treatment.

### 2.7. Defective Immune Response, Dysregulated Energy Metabolism, and Other Targets for HNSCC Therapy

The use of two monoclonal antibodies against programmed cell death 1 (PD-1) was a practical success in overcoming an abnormal immune response of HNSCC cells. Cancer cells produce excessive PD-1 ligands (PD-L1/2), which binds to PD-1 receptors on the surface of T-cells. As a result, T-cell activity, proliferation, cytokine secretion, and overall survival are all affected [87,88]. Pembrolizumab is an FDA-approved IgG4-κ humanized monoclonal antibody against PD-1, activating the immune response [89,90]. Another IgG4 antibody, nivolumab, was also approved to treat HNSCC [91].

Otto Warburg observed specific energy metabolism in cancer cells using glycolysis and fermentation, despite access to oxygen [92]. Nowadays, we have a much better understanding of the so-called Warburg effect. Glycolysis, glutaminolysis, NAD synthesis, tricarboxylic acid cycle, mitochondrial activity, changes in intra- and extracellular pH, lipid and amino acid metabolism, and control of master regulators of energy metabolism such as c-Myc, HIF-1α, Akt, or sirtuins are examples of metabolic targets [93,94,95]. Some commonly used chemotherapeutics target metabolism (e.g., methotrexate - folic acid metabolism) are registered for non-cancer purposes and used in antitumor procedures (e.g., metformin related with glucose metabolism), or are in clinical trials (e.g., AZD-3965 inhibiting lactate transporter MCT1) [96]. The reorganization of cancer cells’ metabolism cooperates with other molecular abnormalities and should be considered an adjuvant therapy in most cases.

**Figure 1:**
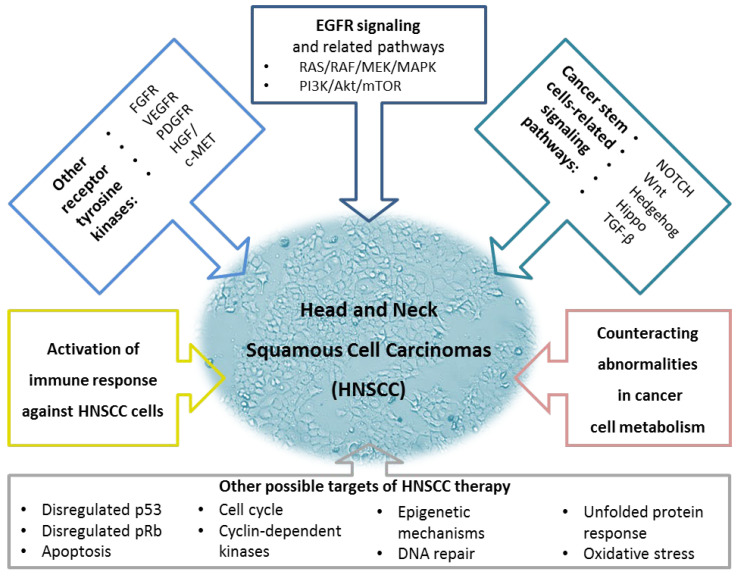
A summary of possible therapeutic targets for Head and Neck Squamous Carcinomas. The upper part of the figure represents essential signaling pathways. The two major new features of cancer cells, changes in immune system response and energy metabolism, are mentioned in the middle. The lower part of the figure demonstrates other possible targets of HNSCC therapy. The figure was created using information from the references given in Section 2 (Molecular Targeted Therapy of HNSCC). EGFR, epidermal growth factor receptor; FGFR, fibroblast growth factor receptor; HGF/c-MET, hepatocyte growth factor/mesenchymal-epithelial-transition factor; MAPK, mitogen-activated protein kinase; mTOR, mammalian target of rapamycin; PDGFR, platelet-derived growth factor receptor; PI3K, phosphoinositide 3-kinase; TGF-β, transforming growth factor-β; VEGFR, vascular endothelial growth factor receptor.

Figure 1 represents the targets discussed in Section 2, enriched with other possible targets of HNSCC therapy [97,98,99,100,101,102,103].

## 3. Attempts at Combinatorial Targeted Therapy

Previously, different molecular targets for HNSCC therapy were demonstrated. Often, the efficacy of the proposed monotherapy could have been greater. Although multi-approach treatment based on standard chemotherapeutics and/or radiation improved the outcomes of HNSCC patients, side effects and overall survival still need attention. Hence, some examples of combinatorial inhibition of molecular targets, primarily signaling pathways controlling HNSCC growth, are presented. This concept is based on the possibility of reprogramming communication between the crucial pathways and, as a result, attenuating cancer cells.

### 3.1. EGRF in the Center of Attention

The first registered molecular targeted therapy for treating HNSCC was EGFR inhibition. That is probably why many reports show attempts to co-target EGFR and other EGFR signaling elements or related signaling pathways. It is also essential, due to acquiring resistance to single EGFR-based targeted therapy [104].

A computer analysis of data from HPV-negative HNSCC samples revealed that *EGFR* amplification and *PI3KCA* mutation (gene encoding p110α catalytic domain of PI3K) were found in more than half of the HNSCC cases, and the active status of the EGFR pathway can help to predict response to the PI3K inhibitor [105]. The co-existence of such molecular abnormalities could require concurrent inhibition of EGFR and PI3K signaling. In six-week-old female NMRI-nu mice (nu/nu) injected with CAL33 mutant cells into the floor of the mouth, EGFR inhibition (via cetuximab) and PI3K inhibition (via buparlisib) were tested as dual treatment or in combination with additional radiotherapy. When compared to an untreated control group and three groups receiving monotherapy, double and triple therapy resulted in greater tumor inhibition [106]. Furthermore, irreversible inhibitors of the tyrosine kinase domain (found, for example, in EGFR structure) were used in conjunction with the PI3K small molecule inhibitor, HS-173. In HNSCC patient-derived cell lines, the combination of afatinib and HS-173 demonstrated synergy not seen in other cases [107].

Additional inhibition of PI3K downstream effectors improved the anticancer effects of drugs targeting EGFR. Partly synergistic effects were shown for co-treatment of SC263 and SCC22b cetuximab-sensitive cell lines and acquired cetuximab-resistant cell lines (SC263-R and SCC22b-R) exposed to combinations of cetuximab and MK2206-pan-Akt inhibitor [108]. Furthermore, the mTOR inhibitor, temsirolimus, combined with cetuximab significantly and synergistically affected the growth of the orthotopic xenograft model of HNSCC [109]. In another study, HNSCC patient-derived xenograft models treated with cetuximab and AZD8055 mTORC1/2 inhibitor showed a greater reduction in tumor growth than cetuximab alone [110].

Signals from EGFR activation are transmitted via RAS kinase to both the PI3K/Akt/mTOR and the RAS/RAF/MAPK pathways. In the HNSCC cell line, UMSCC74B and O28, afatinib inhibited EGFR and PI3K/Akt signaling and simultaneously induced the MEK/MAPK part of RAS signaling. Furthermore, in the same experimental model, the MEK inhibitor, PD0325901, inhibited ERK phosphorylation but, in parallel, enhanced the phosphorylation of Akt and mTOR. When these two molecules were combined, both pathways were inhibited, suppressing synergistically cancer cell proliferation and survival [111]. Synthetic lethality screens using shRNA libraries identified these two targets as promising HNSCC treatment [112]. However, in research on six *PIK3CA*-amplified, PI3K inhibition-resistant HNSCC cell lines, dual inhibition of PI3K and MEK (by HS-173 and trametinib, respectively) displayed synergistic responses only in UM-SCC-69 and UM-SCC-108 cells [113].

Resistance to EGFR-targeted therapy in HNSCC cells could be overcome by simultaneously inhibition of other RTK. Although SCC147 and BICR16 FGFR-amplified cell lines were resistant to an AZD4547 FGFR inhibitor, dual inhibition with addition of gefitinib resulted in synergistically reduced proliferation [48]. The same positive results were obtained when combining gefitinib with the FGFR inhibitor, BGJ398 [114]. Pre-clinical research on cell culture and animal models targeting VEGF (via bevacizumab) in conjunction with cetuximab, augmented antitumor activity, and this pathway combination should be further clinically evaluated [51]. When combined with erlotinib, bevacizumab improved clinical outcomes in some patients with R/M HNSCC [52]. Cetuximab was also used in HNSCC along with sunitinib (a multikinase inhibitor that blocked VEGFR and PDGFR) and irradiation in CAL33 cells growing as orthotopic xenografts in nude mice. This triple combination approach effectively halted tumor growth [115]. Concurrent inhibition of EGFR (by gefitinib) and HGF/c-MET (via crizotinib or SU11274) significantly affected HNSCC cell line proliferation, invasion, and wound healing compared to individual inhibitors. The same findings were made in female C.B-17/IcrHsd-scid mouse xenograft models [116]. Finally, a JAK1/STAT pathway inhibitor (JAK1i) combined with cetuximab as a post-radiotherapy treatment improved anticancer results in the UM-SCC-1 and UM-SCC-5 HNSCC cell lines [117].

Signaling pathways involved in the control of the embryogenesis, proliferation, and differentiation of organisms, controlling stem cells—and in pathologic conditions, also CSC—were thought to be relevant in the development of HNSCC. Therefore, co-inhibiting them with EGFR should be beneficial for HNSCC treatment. CAL 27 and HN6 cells were treated with erlotinib and PF-03084014, a selective inhibitor of NOTCH signaling, which reduced proliferation and evasion compared to erlotinib alone. Furthermore, such dual treatment revealed synergy in the inhibition of the PI3K/Akt pathway. This combination significantly affected tumor growth in male BALB/c-nu mice in vivo [118]. Erlotinib was recently combined with PRI-724, an inhibitor of Wnt/β-catenin signaling, in CAL 27 and FaDu HNSCC cells. There was a synergistic reduction in cell viability and migration, as well as disruption in cell cycle progression and quite intense induction of apoptosis [119]. Furthermore, in commercial HN5 and FaDu cells, as well as 20 different HNSCC patient-derived cell lines, a combination of cetuximab and vismodegib (inhibitor of the Hh pathway) confirmed the benefits of EGFR and Hh co-inhibition [120]. TGF-1β is highly expressed in HPV-negative HNSCC patients but not in HPV-positive cases [121]. Thus, similarly to EGFR co-inhibition with the Hippo/Yap pathway, the concurrent attenuation of the EGFR pathway and activity of TGF-β signaling is worth noting [121,122].

EGFR can be inhibited together with other targets. Inhibition of EGFR using gefitinib induces the activity of proinflammatory NF-κB signaling via the mechanism of intracellular pathway crosstalk and compensatory mechanisms [123,124]. Combining gefitinib with CmpdA (inhibitor of I-Kappa-B kinase-beta (IKKβ) regulatory subunit of NF-κB pathway) [123] or Bay117085 (IκBα kinase and NF-κB inhibitor) [124] in pre-clinical models suggests that this EGFR-NF-κB axis inhibition is an excellent alternative approach for HNSCC treatment. Reactivation of immune response against HNSCC cells by inhibiting PD-1 receptor and PD-L1/2 ligands is an FDA-approved treatment. As a result, the effects of cetuximab application are frequently compared to pembrolizumab, shown, for instance, in [125,126,127]. Taking a step forward, a study of the concurrent use of cetuximab and pembrolizumab was included also in a phase II clinical trial for patients with R/M HNSCC. Notably, 15 of the 33 patients achieved a partial response, and there were no treatment-related deaths, indicating that such a combination is promising and requires further investigation [128].

HNSCC cells are an excellent illustration of the Warburg effect because they are heavily dependent on glucose metabolism, and EGFR signaling promotes glucose uptake and utilization in aerobic glycolysis. In vitro combination of 2-deoxyglucose (2-DG) inhibiting glycolytic pathway with erlotinib in FaDu, CAL 27, and SQ20B HNSCC cell lines additionally reduced their viability. However, in vivo, this effect was counteracted by tumor-rescue autophagy induction [129]. After all, the modulation of altered energy metabolism deserves further investigation. Progress in HNSCC therapy may be observed through combinations of, e.g., cetuximab with cyclin-dependent kinase inhibitors [130]. Moreover, HNSCC radiotherapy aims to cause DNA breaks, which should result in cancer cell death. During the activation of DNA repair systems, some issues may arise. DNA repair is promoted by EGFR signaling and poly (adenosine diphosphate-ribose) polymerase-1 (PARP1). Thus, cetuximab was tested in vitro and in mice xenograft models with olaparib-PARP inhibitor, and radiation. This triple combination has demonstrated improvements in HNSCCs’ responses to treatment and justified launching clinical trials [131]. Demethoxycurcumin (DMC) suppressed HNSCC through G2/M-phase arrest and cell apoptosis, where downregulation of X-chromosome-linked IAP (XIAP) was crucial. Furthermore, blocking EGFR activation with gefitinib and XIAP activation with DMC significantly improved gefitinib’s antiproliferative activity [132]. Apoptosis was also excessively induced via a combination of cetuximab and R763 pan-Aurora kinase inhibitor, with additional cell cycle checkpoints activation [133]. Finally, chemicals that target epigenetic dysregulations may be considered as well. For instance, in CAL 27 and FaDu cells, erlotinib was combined with histone lysine demethylases (KDM) inhibitors, namely, ML324 (inhibitor of KDM4) and GSK-J4 (inhibitor of KDM6). Both combinations exposed partial synergistic effects in reducing HNSCC cell viability and synergy in the induction of apoptosis, except ML324 in FaDu cells due to the intense apoptosis already for single use of erlotinib and ML324. Moreover, these combined treatments reduced the expression of the anti-apoptotic survivin, the cell cycle-controlling cyclin D1, and increased the expression of the tumor suppressive factor p21 [134].

Many options for improving response to EGFR inhibition-based targeted therapy have been explored, but the task still needs to be completed. Overcoming EGFR monotherapy resistance may require patient-specific signaling signature (PaSSS) analyses to develop PaSSS-based drug combinations suitable for altered signaling networks in HNSCC [135].

Combinations of drugs inhibiting EGFR with another molecularly targeted treatment, discussed in Section 3.1, are summarized in Table 1.

### 3.2. Combined Molecular Targeted Therapy Omitting EGFR

Studies that do not include direct EGF receptor inhibition provide an alternative approach to molecularly targeted combinatory therapy of HNSCC. Several examples are provided below.

RAS kinase has been identified as a critical component of intracellular signal transduction. Tipifarnib, a farnesyltransferase inhibitor, was combined with the inhibition of RAS-related ERK and PI3K kinases, which improved the response of HNSCC cell lines to tipifarnib. Tipifarnib-induced, epithelial-to-mesenchymal transition was blocked in the case of ERK inhibition [136].

PI3K signaling, co-inhibited with FGFR (by alpelisib and erdafitinib, respectively), had a significant effect on tonsillar and base-of-tongue squamous cell carcinoma (TSCC/BOTSCC), regardless of *PIK3CA* and *FGFR3* mutations [137]. Inhibition of PI3K by HS-173 combined with KDM4 or KDM6 inhibitors disrupted CAL 27 and FaDu cells, similarly to previously mentioned combinations of those epigenetic modifiers with erlotinib. Additionally, in hypopharyngeal cancer FaDu cells, HS-173 and KDM inhibitors had a strong synergistic effect on cell viability [134]. The PI3K pathway was also co-targeted with NOTCH signaling, giving progressive effects [138,139].

Inhibition of PI3K by HS-173 was tested in conjunction with the Wnt/β-catenin inhibitor PRI-724. This drug combination worked in a highly synergistic way to reduce FaDu cell viability and to interrupt cell cycle progression by accumulating cells in the S and G2/M phases. It was also followed by a reduced migration rate, concerning both FaDu and CAL 27 cell lines [119]. In the same study, PRI-724 was combined with vismodegib (a Hh signaling inhibitor), resulting in decreased cell proliferation, synergistic reduction of cell migration, and significant mRNA down-regulation of the Oct4 stemness marker. Wnt signaling is especially worth attention because concomitant inhibition of its canonical variant (via PRI-724) or canonical and non-canonical variants (via IWP-O1) with an Akt kinase inhibitor was efficient in reducing survival of tongue SCC cell lines CAL 27 and SCC-25, as well as tongue metastatic BICR22 cells [140]. Cell viability measured based on ATP content was significantly reduced after Akt inhibition and, in part, after a combination of Wnt and Akt inhibitors. Interestingly, Wnt pathway inhibitors significantly reduced glucose utilization and lactate production by tongue SCC cells. This effect on cancer cell energy metabolism was further confirmed and improved by testing Wnt signaling inhibitors together with glycolytic inhibitors 2-DG and lonidamine [141].

Inhibition of the PI3K/Akt/mTOR pathway can be followed by the concurrent use of chemicals that affect cell cycle progression. In this regard, cooperation between everolimus, which targets m-TOR kinase, and LY2835219, which inhibits cyclin-dependent kinase (CDK) 4/6, resulted in synergistic tumor growth inhibition and should undergo further therapeutic investigation [142]. In another study, both HPV-positive and HPV-negative HNSCC cells exhibit frequent synergy for CDK 4/6 and PI3K co-inhibition, as well as a slight synergy for PI3K and FGFR inhibitors [143]. Finally, when compared to single inhibitors, the BYL719 inhibitor of PI3K and BMN-673 inhibitor of PARP showed synergistic effects in TSCC/BOTSCC cells, which is a practical example of preventing DNA repair in HNSCC cells [144].

Exemplary combinations of molecular targets, other than EGFR, are summarized in Table 2.

**Figure 2:**
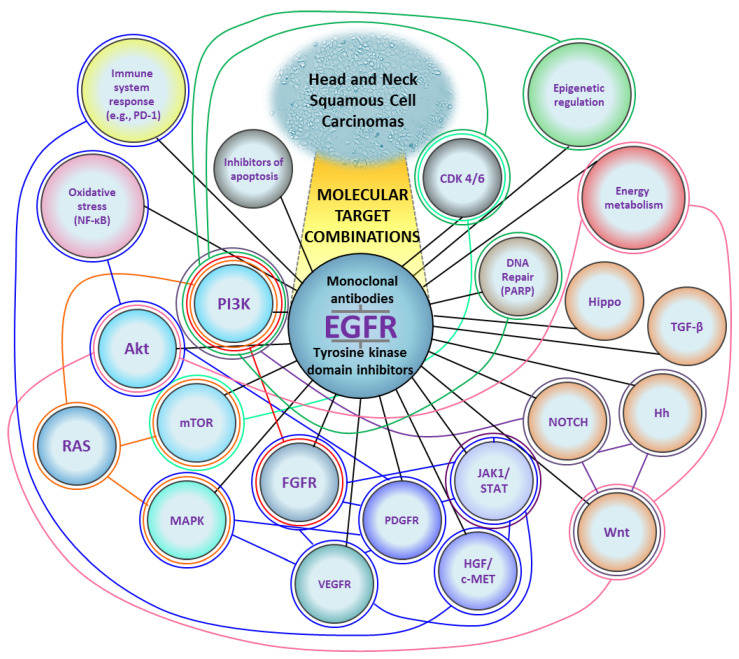
A diagram of molecular targets for the treatment of Head and Neck Squamous Cell Carcinomas. Black lines represent combinations of EGFR inhibitors with other targets. Colored lines represent combinations that do not include EGFR. Based on the data cited in Section 3 (Attempts at Combinatorial Targeted Therapy). CDK 4/6, cyclin-dependent kinase 4/6; EGFR, epidermal growth factor receptor; FGFR, fibroblast growth factor receptor; HGF/c-MET, hepatocyte growth factor/mesenchymal-epithelial-transition factor; Hh, Hedgehog; JAK1/STAT, Janus kinase 1/signal transducer and activator of transcription; MAPK, mitogen-activated protein kinase; mTOR, mammalian target of rapamycin; NF-κB, nuclear factor kappa-light-chain-enhancer of activated B cells; PARP, poly (adenosine diphosphate-ribose) polymerase-1; PD-1, programmed cell death 1; PDGFR, platelet-derived growth factor receptor; PI3K, phosphoinositide 3-kinase; TGF-β, transforming growth factor-β; VEGFR, vascular endothelial growth factor receptor.

Figure 2 shows a schematic representation of the molecularly targeted treatment combinations described in Section 3.

## 4. Limitations of Targeted Therapy

The development of cancer-targeted therapy is intended to increase patients’ positive outcomes. The achievements in this respect are undoubtedly significant. However, resistance to single-agent therapies is also observed, like in the previously mentioned inhibition of EGFR. Even molecularly targeted co-treatment can be insufficient in many cases of pre-clinical and clinical research [145,146,147]. Co-treatment of cancer with molecularly targeted drugs can help avoid resistance but, due to a considerable number of possible combinations, there is a need for rational compositions of medications based on a deep understanding of mechanisms associated with therapy resistance [148].

Ideal, tumor-specific therapies should be neutral to non-cancerous tissues. Unfortunately, many molecularly targeted chemicals are also active on wild-type targets and open the way for developing the on-target side effects, also called target-related or mechanism-based side effects [149,150]. For instance, inhibition of EGFR affects tissues normally dependent on EGF signals, causing skin dryness, acneiform rashes, and skin infections [151]. Moreover, EGFR inhibitors favor the occurrence of nail pathology (e.g., paronychia), problems with hair (folliculitis, follicular necrosis, alopecia), and mucosal changes [152]. Drugs targeting PI3K/Akt/mTOR pathway were reported to cause hyperglycemia and hyperlipidemia, bone marrow suppression (particularly – anemia, thrombocytopenia, and neutropenia), stomatitis, hepatotoxicity, and pneumonitis [153]. Of course, off-target side effects (adverse effects as a result of modulation of other targets) can occur, but they are primarily a problem of standard therapy [150].

Another complication of targeted therapy is the high costs of these drugs, making them inaccessible to many clinics and for all patients [154,155]. The use of selective biomarkers to select patients with molecular changes being a target for a particular drug may slightly reduce the costs but not influence the treatment compound’s high price [156].

Some molecular targets in cancer cells are called undruggable. Difficulties are caused, e.g., by a lack of defined ligand-binding pockets or complications with a description of 3D structures [157]. KRAS proto-oncogene belongs to this group of targets. Nowadays, sotorasib—a small molecule that selectively and irreversibly targets *KRASG12C*-mutated cells—shows valuable anticancer activity in patients with heavily pretreated advanced solid tumors harboring this mutation [158]. Thus, at least partly, undruggable targets can be transformed into new therapeutic options.

An additional issue concerns complex, multifaceted, and interrelated barriers to clinical trial enrollment. Clinical studies need, e.g., more staff and financial support, better inter-departmental cooperation, and the removal of unnecessary regulatory barriers [159].

Precision medicine and personalized therapy are critical for implementing “tailored treatment” [160,161,162]. The majority of the treatment options discussed in Section 3 can be used to disrupt cancer cell communication. Thus, normalizing molecular signaling is thought to be the next step in the development of anticancer treatment, where even vitamin D concentration and receptor (VDR) expression may influence HNSCC patients’ therapeutic outcomes [163]. It can also help overcome some complications connected with targeted therapy. The fundamentals of this concept are discussed below.

## 5. Anakoinosis-Based Cancer Therapy

The word, “Anakoinosis,” is derived from Ancient Greek and means “Communication”. Cancer cells appear to form an extensive communication network with each other and with non-cancer cells. Conventional cancer therapy aims to induce cancer cell death, e.g., by promoting DNA damage and subsequent apoptosis [164]. To prevent tumor recurrence and metastasis, there is need to reorganize pathological homeostasis created by cancer cells. The previously discussed combined molecularly targeted treatment may be considered a good step forward because inhibiting more than one signaling pathway, or signaling pathway(s), and another tumorigenic mechanism can disorganize cancer cells.

The anakoinosis-based approach to tumor treatment employs different mechanisms to affect cancer cells. It includes metronomic chemotherapy, drug repurposing, oxidative stress, metabolic and transcriptional modulators, epigenetic drugs, and immune response activators [165,166,167].

Metronomic chemotherapy refers to the use of a minimum biologically effective dose of anticancer compounds administered as a continuous dosing scheme in contrast to treatment cycles separated by long breaks [166]. Drug dose reductions can alter a drug’s detailed mechanism of action in cancer cells. For instance, etoposide (topoisomerase II inhibitor) at a concentration of 50 µM was found to induce rapid caspase-3-mediated apoptosis in myeloid leukemia cell lines, which is, unfortunately, also a significant promoter of wounded tissue regeneration and post-therapy cancer repopulation. In turn, a concentration of 0.5 µM was found to induce morphological and functional granulocytic differentiation and caspase-2-dependent cell death [168].

In the context of anakoinosis, drug repurposing is the process of selecting appropriate combinations of approved drugs with pro-anakoinotic activity profiles to improve therapeutic effects and, ideally, achieve complete tumor remission [167]. Pioglitazone, used for the treatment of diabetes, is an agonist of the peroxisome-proliferator-activated receptor, α/γ (PPARα/γ), that modulates the transcription of genes involved in the control of energy (glucose and lipid) metabolism. It also can normalize cancer cells communication [169,170]. Pioglitazone is often administered in combination with metronomic chemotherapy and a COX-2 inhibitor [165,171]. Indeed, COX-2 inhibitors, in addition to their anti-inflammatory properties, can inhibit aggressive cancer cells, such as glioblastoma-derived cells, through a mechanism related also to Wnt/β-catenin signaling attenuation [172]. Moreover, natural compounds such as lichen-derived depsides and depsidones act against the interconnection of the NF-κB, Nrf2, and STAT3 signaling pathways, which can be considered an anakoinosis-related treatment [173]. Furthermore, conjugates of natural oleanolic acid and synthetic, nonsteroidal, anti-inflammatory drugs, e.g., aspirin, indomethacin, and diclofenac, have demonstrated therapeutic activity against pancreatic and hepatocellular carcinoma cells, which was linked to changes in NF-κB and Nrf2 signaling [174,175,176,177,178]. Changes in cancer cell communication are also associated with interference in signaling pathways related to cell differentiation, stemness, and proliferation. For instance, targeting the NOTCH pathway may be useful [179], but Wnt and Hh pathways should not be omitted.

Some typical anakoinosis-based therapies are under evaluation. A patient with acute myeloid leukemia after allogeneic hematopoietic stem cell transplantation, who was refractory to one cycle of azacytidine (DNA methyltransferase inhibitor), received a low metronomic dose of azacytidine plus pioglitazone and all-trans-retinoic acid. As a result, a total remission was observed [180]. The same triple chemical combination effectively treats leukemia and cutaneous leukemic infiltrates [181]. Even when triggered by infection, proper immune system stimulation is a promising method for normalizing the organism’s response to cancer cells [182]. Reported anakoinosis-based treatment sometimes contains, e.g., dexamethasone (steroid drug) and anti-inflammatory etoricoxib [183,184]. Additionally, as previously stated, vitamin D can be helpful in the treatment of a variety of diseases [163].

**Figure 3:**
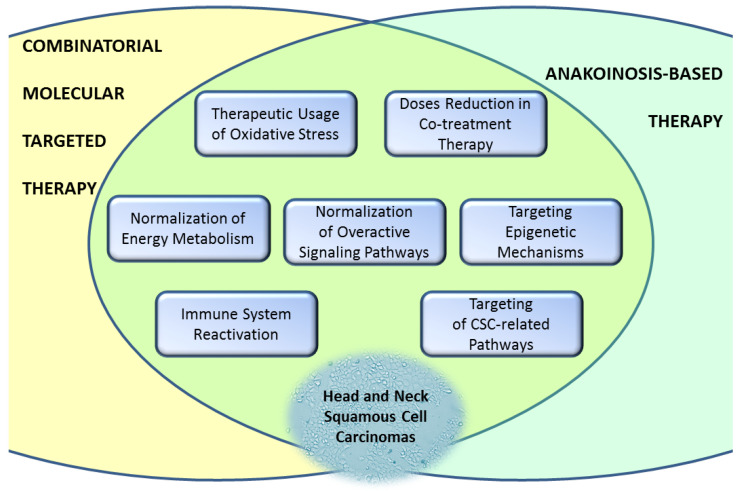
The similarities between combinatorial molecular targeted therapy and anakoinosis-based therapy are significant for future treatment procedures. CSC, cancer stem cells.

Hopefully, the concept of anakoinosis-based therapeutic procedures will be evaluated and introduced into clinics for HNSCC in the future. Current studies exploring combined molecular therapy of head and neck tumors are partly related to anakoinosis. It is most commonly seen in combinations with CSC-related pathways (NOTCH, Wnt/β-catenin, Hedgehog), PD-1 inhibitors, modulators of energy metabolism and epigenetic modifications, or simply combinations of signaling pathways, which inhibition tend to stimulate related intracellular signaling (Figure 3). A combination of molecularly targeted drugs uses lower doses of active compounds due to the synergistic effect, resulting in a metronomic-like treatment with potentially fewer side effects. The development of this strategy can be accelerated using computational modeling to analyze and predict communicative reprogramming [185].

## 6. Conclusions and Perspectives

**Figure 4:**
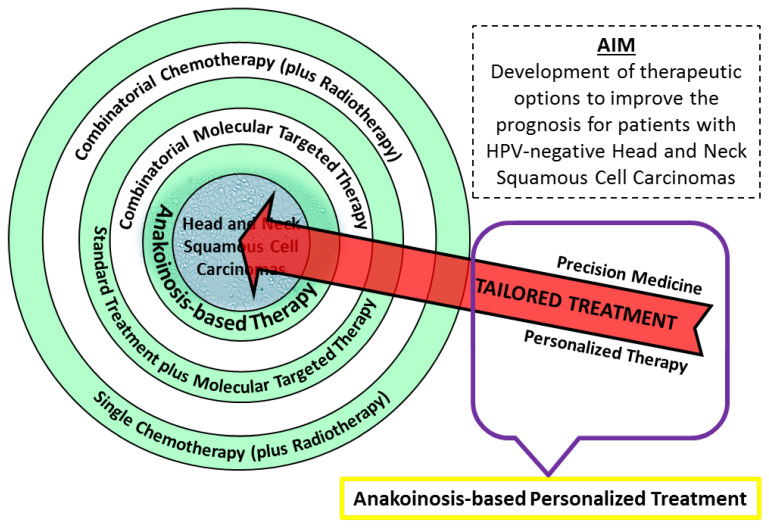
A graphic overview of treatment options for Head and Neck Squamous Cell Carcinoma patients. New ideas include combinatorial molecular targeted therapy and the anakoinosis concept. To implement “tailored treatment,” further advancement in precision medicine and personalized therapy is required. Hopefully, in the future new class of oncological therapy will be created—anakoinosis-based personalized treatment.

Oncological therapy has come a long way since implementing standard chemotherapeutics appropriate for HNSCC tumors, often in combination with radiotherapy. Molecularly targeted therapy continues to be explored to overcome the limitations of standard procedures. Most clinical trials consider the simultaneous use of chemotherapeutics (together with radiation) and novel compounds suitable for molecular targets of particular cancer-type. Combinatorial molecular targeted therapy for head and neck cancer is mostly found in pre-clinical stages. The inhibition of EGF receptors with other targets appears to be a central point of interest, intending to defeat cancer cell resistance to single inhibition of EGFR. This review has discussed exemplary concepts for improving HNSCC therapy by searching for new effective combinations of molecular targets dysregulated in HNSCC. Hopefully, the coming years will bring more exciting reports regarding the development of anakoinosis-based treatments, meaning the normalization of cancer cells’ communication to make them sensitive to death-inducing compounds or simply killing cells through various mechanisms. Metronomic, low-dose combined therapy, drug repurposing, stemness modulators, and others will likely improve the effects against HNSCC tumors, especially in HPV-negative cases with worse prognoses (Figure 4).

By developing personalized molecular therapy, in which the strategy of eradicating tumor is precisely created for a particular patient (tailored treatment), we can reach a new level of oncological targeted therapy—anakoinosis-based personalized treatment.

## Figures and Tables

**Table 1:** Summary of the effects of targeted therapy against EGFR combined with a selective inhibitor of another target.

Second Target in Combination	Combination EGFR inh. + Second inh.	General Effect and Experimental Model *	Reference
PI3K (phosphoinositide 3-kinase)	cetuximab + buparlisib (+ radiotherapy)	improved tumor inhibition via double/triple combination (in vivo)	[106]
	afatinib + HS-173	synergistic anticancer activity (ex vivo)	[107]
Akt kinase	cetuximab + MK2206	partly synergistic anticancer activity (in vitro)	[108]
mTOR (mammalian target of rapamycin)	cetuximab + temsirolimus	synergistic tumor growth inhibition (in vivo)	[109]
	cetuximab + AZD8055	improved reduction of tumor growth (in vivo)	[110]
MAPK (mitogen-activated protein kinases)	afatinib + PD0325901	prevention of single-compound-related resistance and synergistic reduction of cancer cells survival (in vitro)	[111]
FGFR (fibroblast growth factor receptor)	gefitinib + AZD4547	synergistic reduction of cell proliferation (in vitro)	[48]
	gefitinib + BGJ398	synergistic tumor growth inhibition (in vivo)	[114]
VEGFR (vascular endothelial growth factor receptor)	cetuximab + bevacizumab	improved anticancer activity (in vitro) and reduction of tumor growth (in vivo)	[51]
	erlotinib + bevacizumab	better outcomes in patients with R/M HNSCC ** (clinical trial)	[52]
VEGFR and PDGFR (platelet-derived growth factor receptor)	cetuximab + sunitinib + radiotherapy	improved reduction of tumor growth (in vivo)	[115]
HGF/c-MET (hepatocyte growth factor/mesenchymal-epithelial- transition factor)	gefitinib + crizotinib or gefitinib + SU11274	reduced cell proliferation, migration and invasion (in vitro), and reduced tumor growth (in vivo)	[116]
JAK/STAT (Janus kinase/ signal transducer and activator of transcription)	cetuximab + JAK1i	improved anticancer activity (in vitro)	[117]
NOTCH	erlotinib + PF-03084014	reduced proliferation and invasion, synergy in inhibition of PI3K pathway (in vitro), and improved reduction of tumor growth (in vivo)	[118]
Wnt (canonical)	erlotinib + PRI-724	synergy in reduction of cell proliferation and migration, accelerated apoptosis (in vitro)	[119]
Hedgehog	cetuximab + vismodegib	improved anticancer activity (in vitro and ex vivo)	[120]
TGF-β (transforming growth factor-β)	cetuximab + antibody against TGF-β	improved reduction of tumor growth (in vivo)	[121]
NF-κB (nuclear factor kappa-light-chain-enhancer of activated B cells)	gefitinib + CmpdA	improved anticancer activity (in vitro)	[123]
	gefitinib + Bay117085	improved anticancer activity (in vitro) and reduced tumor growth (in vivo)	[124]
PD-1/PD-L1 (programmed cell death 1/programmed cell death ligand 1)	cetuximab + pembrolizumab	better outcomes in patients with R/M HNSCC ** (clinical trial)	[128]
Glycolysis	erlotinib + 2-deoxyglucose	reduced cell viability (in vitro) and loss of effects due to tumor-rescue autophagy induction (in vivo)	[129]
CDK (cyclin-dependent kinase)	cetuximab + palbociclib	synergistic viability reduction (in vitro)	[130]
PARP1 (poly (adenosine diphosphate-ribose) polymerase-1))	cetuximab + olaparib + radiotherapy	improved anticancer activity (in vitro) and reduced tumor growth (in vivo)	[131]
XIAP (X-linked inhibitor of apoptosis protein)	gefitinib + demethoxycurcumin	activation of cell cycle arrest and induction of apoptosis (in vitro)	[132]
Aurora kinase	cetuximab + R763	accelerated induction of apoptosis and cell cycle checkpoint activation (in vitro)	[133]
KDM (histone lysine demethylase)	erlotinib + ML324 (inhibitor of KDM4) or erlotinib + GSK-J4 (inhibitor of KDM6)	synergistic inhibition of cell viability and activation of apoptosis (in vitro)	[134]

* clinical trial, research studies on human participants; ex vivo, experiments using primary cancer cell cultures derived from patients; in vitro, experiments using cancer cell cultures; in vivo, animal experiments (xenograft models); ** R/M HNSCC, recurrent and metastatic head and neck squamous cell carcinoma.

**Table 2:** Summary of the effects of targeted therapy combinations omitting EGFR.

First Molecular Target /Drug Name/	Second Molecular Target /Drug Name/	General Effect and Experimental Model *	Reference
RAS farnesylation /tipifarnib/	PI3K pathway (phosphoinositide 3-kinase) /alpelisib/	increased sensitivity of cancer cells to tipifarnib (in vitro)	[136]
	MAPK pathway (mitogen-activated protein kinase) /SCH772984/		
PI3K /alpelisib/	FGFR (fibroblast growth factor receptor) /erdafitinib/	synergistic reduction of cell viability (in vitro)	[137]
PI3K /HS-173/	KDM4 (histone lysine demethylase 4) /ML324/	synergistic inhibition of cell viability and induction of apoptosis (in vitro)	[134]
	KDM6 (histone lysine demethylase 6) /GSK-J4/		
PI3K /GSK2126458/	NOTCH /*NOTCH1* mutant cells/	improved and predicable response to PI3K pathway inhibitors (in vitro)	[138]
mTOR (mammalian target of rapamycin) /ridaforolimus/	NOTCH (MK-0752)	partial response to treatment, problems with side effects in maximum tolerated dose (clinical trial)	[139]
mTOR /everolimus/	CDK 4/6 (cyclin-dependent kinase 4/6) /LY2835219/	improved anticancer activity (in vitro) and synergistic tumor growth inhibition (in vivo)	[142]
CDK 4/6 /PD-0332991/	PI3K /BYL719/	synergistic reduction of cell viability (in vitro)	[143]
	FGFR /NJ-42756493/		
PARP (poly (adenosine diphosphate-ribose) polymerase)) /BMN-673/ (+ radiotherapy)	PI3K /BYL719/ (+ radiotherapy)	synergistic reduction of cell viability by drugs combination, lack of additional effect of irradiation (in vitro)	[144]
Wnt (canonical) /PRI-724/	PI3K /HS-173/	synergy in reduction of cell viability and migration rate (in vitro)	[119]
	Hedgehog /vismodegib/		
Akt kinase /Akt kinase inhibitor X/	Wnt (canonical) /PRI-724/	reduced viability of cells growing in 2D and 3D culture, decreased glycolytic activity-glucose intake and lactate release (in vitro)	[140]
	Wnt (canonical and non-canonical) /IWP-O1/		
Glycolysis /2-deoksyglucose/ and /lonidamine/	Wnt (canonical) /PRI-724/	reduced cell viability, decreased glycolytic activity—glucose intake and lactate release (in vitro)	[141]
	Wnt (canonical and non-canonical) /IWP-O1/		

* clinical trial, research studies on human participants; in vitro, experiments using cancer cell cultures; in vivo, animal experiments (xenograft models).
